# Supplementary material for: Genome reduction occurred in early Prochlorococcus with an unusually low effective population size
Source: ISME J. 2024 Jan 10;18(1):wrad035. doi: 10.1093/ismejo/wrad035 (PMC10837832; doi:10.1093/ismejo/wrad035)
Supplement: SI_20231220_wrad035 [file si_20231220_wrad035.pdf]

Supplementary Information for

Genome reduction occurred in early *Prochlorococcus* with an unusually low effective population size

Hao Zhang^, Ferdi L. Hellweger^, Haiwei Luo

^Co-first author

Contact: Haiwei Luo (haiweiluo@cuhk.edu.hk)

This file includes:

- **Methods**
- **References**
- **Fig. S1 to S5**

Methods

Agent-based model building

We used an updated version of the IAM model presented previously [1]. The model code and sample input files are deposited in the online GitHub repository (https://github.com/luolab-cuhk/Prochl-ABM).

To support the simulations presented here, an additional nonsynonymous mutation penalty sub-model (no. 8, see Table S1 in Hellweger et al., 2018 and associated text and references) was implemented in the code. This was done for the following reason. In the previous version, nonsynonymous mutations were assumed to be either functionally neutral or deleterious, with the probability of being deleterious dependent on the amino acid chemical distance. Deleterious mutations were assumed to be lethal, which was done to exclude them from the population, otherwise they would gradually lower the population growth rate (i.e. Muller's Ratchet). This approach is realistic for constant and large effective population size (N_e), as was the case in the previous model application. The previous application focused on the role of nutrient limitation in shaping the genome, and not the effect of N_e on d_N/d_S or d_R/d_C. However, this approach does not explicitly simulate purifying selection and the effect of N_e on that, which is the focus of the present model application. Here, the deleterious mutations have to "stay in the population" (i.e., we cannot simply remove them by making the mutation fatal) so that they can be outcompeted, which will be a function of N_e.

The new nonsynonymous mutation fitness (previously called penalty) sub-model is as follows. For each nonsynonymous mutation, a selection coefficient (s) is drawn from a

probability distribution. The distribution consists of an exponential distribution below 0 (deleterious mutations) and a uniform distribution above 0 (advantageous mutations) (Fig. S1). The distribution is defined by three parameters, including the fraction of mutations that are deleterious (f_{del}), and the average selection coefficients for the deleterious mutations (s_{pn}) and the advantageous mutations (s_{an}). f_{del} and s_{an} are global parameters. s_{pn} is a function of the amino acid (AA) dissimilarity. Specifically, the s_{pn} for mutations between AA i and j is:

$$s_{pn}(i,j) = s_{base}\frac{d(i,j)}{d_{base}}$$

$s_{pn} = s_{base}$ for $d(i,j) = d_{base}$.

$d(i,j)$ = AA dissimilarity matrix.

The selection coefficients for all occurred mutations affect the growth rate (via the nutrient uptake rate, see previous model description). The model does not explicitly consider any biological constraints on the overall fitness of a cell and to avoid biologically unrealistic increases in fitness (i.e., Darwinian Demon), the growth rate is not allowed to exceed (i.e., is capped at) that of the ancestor in the simulation.

For recombination, selection coefficients of incoming changes/mutations from the donor overwrite those of the recipient in the simulation.

Agent-based model application

Four bacterial species were chosen as the targets for modelling with ABM, including *Prochlorococcus marinus* AS9601 (GCF_000015645), *Bacillus subtilis* NCIB 3610 (GCF_002055965), *Ruegeria pomeroyi* DSS-3 (GCF_000011965), and *Vibrio fischeri* ES114 (GCF_000011805). They each have publicly available data of unbiased global mutation rate

determined by the MA/WGS strategy (3.50×10^{-10}, 3.28×10^{-10}, 1.39×10^{-10}, and 2.07×10^{-10} per site per generation, respectively) [2-5] and they are phylogenetically diverse. Their genome sequences were downloaded from the curated NCBI RefSeq database. We employed Prodigal v2.6.3 [6] to call protein-coding genes on genomic sequence and then generate the genomic feature file (GFF). GFF records the start and end positions of ORFs and was used as the input for genome simulation (see example file in the online GitHub repository).

In our simulations, N_e is a function of the census population size (N_c) at the beginning of the growth period (N_c0) and the number of generations in the dilution step (n_G) as follows:

$$N_e = N_c0 * n_G$$

The factor n_G is another function of the growth rate [k_G, determined by the availability of C ($V_{max}0C$), N ($V_{max}0N$), and P ($V_{max}0P$)] and the dilution time step (d_t) as follows:

$$n_G = \frac{d_t}{(\frac{ln2}{k_G})}$$

All the abovementioned model parameters are deposited in a configuration file, which is required for our simulation (see the example configuration file in online GitHub repository). The default parameters for the nonsynonymous mutation fitness model were calibrated for *Prochlorococcus marinus* AS9601, so that $d_N/d_S = 0.05$ for $N_e = 10^7$. In theory, the stochastic model generates large differences between runs due to chance beneficial mutations and population sweeps. We therefore ran each simulation five times with different seed number (RS_i and RS_j in the configuration file) to reduce the random error and perform statistical tests. As each run samples 50 genome sequences, we obtained a total of 50×5 genome sequences under each putative N_e. For test, an additional run was performed to sample 500 individual

genomes for each N_e. By doing this, we found that the d_N/d_S and d_R/d_C values of *Prochlorococcus* populations remain largely consistent irrespective of the sample size (Fig. S4). To ensure that we have simulated a sufficient number of generations, for each run we sampled genomes at a fixed simulated time interval (5 million years; My). We found that both the d_N/d_S and d_R/d_C ratios of the *Prochlorococcus* populations reach plateau in the early stage at ~50 My (Fig. S2). Longer run time further increases the value of d_N, d_S, d_R, and d_C in the population, but the d_N/d_S and d_R/d_C ratios remain constant.

To simulate the genome sequences under different growth rates, we revised the growth rate-associated parameters ($V_{max}0C$, $V_{max}0N$ and $V_{max}0P$) to 10%, 50% and 200% of the default. According to above equations, we revised the dilution time step (d_t) to 1000%, 200% and 50% of the default, respectively, to keep a constant N_e in these simulations. For 10% growth rate simulations, we also increased the simulation time (*tend* in the configuration file) to ensure accumulating a sufficient number of substitutions in their genome sequences. Whereas the model includes various effects of mutations, e.g., changes to the C, N, and P requirements of DNA and protein pool, the present application only considers the effect of nonsynonymous mutations on protein function.

Our simulations were performed with a wide range of N_e values, spanning from 1e3 to 1e7. Notably, simulations with 1e7 necessitate a minimum of three days of wall-time to complete a single run using 16 CPUs in an HPC system. Further increases of N_e would exponentially extend the computational time. For example, the simulation with 1e8 requires ~45 days of wall-time to complete a single run using 16 CPUs in the HPC system. Given that our conclusion is well supported with the simulations up to 1e7 and that the model is

computationally very demanding, we do not anticipate any changes in the conclusions by further increasing the population size.

Genome-wide d_N/d_S and d_R/d_C calculation

We extracted protein-coding gene sequences from simulated genomes with in-house scripts based on GFF instead of using Prodigal to avoid skipping ORFs in which substitutions occurred at start or stop codon. We estimated the d_N and d_S values of each protein-coding gene with the program YN00 in PAML package v4.9e [7], which takes into account of the transition/transversion ratio (ts/tv) bias and the codon usage bias. To obtain the genome-wide d_N/d_S value, let $N_{i(x)}$ and $S_{i(x)}$ be the number of nonsynonymous and synonymous substitutions and let $N_{j(x)}$ and $S_{j(x)}$ be the nonsynonymous and synonymous sites for each gene (x). Let n be the number of genes in the focal bacterial strain. The genome-wide d_N/d_S is computed as:

$$d_N/d_S = (\frac{\sum_1^n N_{i(x)}}{\sum_1^n N_{j(x)}})/(\frac{\sum_1^n S_{i(x)}}{\sum_1^n S_{j(x)}})$$

To estimate the d_R and d_C values for each gene, we employed the software MEGA-CC v10.2.4 [8] to calculate ts/tv and then passed the ratio to the software RCCalculator [9]. The latter recruits two new models, either based on amino acid frequency or based on codon frequency, to correct for G+C content bias. To obtain the genome-wide d_R/d_C value, let $R_{i(x)}$ and $C_{i(x)}$ be the number of radical and conservative nonsynonymous substitutions and let $R_{j(x)}$ and $C_{j(x)}$ be the radical and conservative nonsynonymous sites for each gene (x). Let n be the number of genes in the focal bacterial strain. The genome-wide d_R/d_C is computed as:

$$d_R/d_C = (\frac{\sum_1^n R_{i(x)}}{\sum_1^n R_{j(x)}})/(\frac{\sum_1^n C_{i(x)}}{\sum_1^n C_{j(x)}})$$

Inferring gene gain and loss events along species phylogeny

The gene gain and loss process along phylogeny is commonly reconstructed by reconciliating incongruence between gene tree and species tree. There are multiple reconciliation software tools, which often give different predictions. To find out the tool that fits best our dataset, we benchmarked four tools in common use, including the likelihood-based ALE v0.4 [10] and GeneRax v2.0.4 [11], as well as the parsimony-based AnGST v1.0 [12] and ecceTERA v1.2.4 [13].

Our benchmarking strategy requires user-specified gene tree as "real tree", uses a tool to simulate sequence alignment from the "real tree", constructs initial gene tree from the simulated alignment and generates a reconciled gene tree, and employs the Robinson-Foulds (RF) distance to assess the topological difference between the reconciled tree and the "real tree" (Fig. S5). To reduce the computational cost while keeping the complexity of the input gene tree, we compiled a small-scale dataset containing 1,000 out of the 4,689 pre-identified gene families of *Prochlorococcus* [14] by sorting the gene family size and sampling with a fixed periodic interval (family size interval=4). Since true gene tree for empirical data is not available and since gene tree reconstructed by using reconciliation method is often more accurate than that reconstructed based on molecular sequence alone [10], we reconciled the IQ-TREE-derived gene tree with *Prochlorococcus* species tree using AnGST v1.0 and GeneRax v2.0.4 to generate the "real tree" dataset I and the "real tree" dataset II, respectively (Fig. S5). The implementation of the two datasets helps to reduce the bias in benchmarking analysis towards either the maximum likelihood (ML) or the maximum parsimony (MP) algorithm. The *Prochlorococcus* species tree and gene family tree used in this step were all

adapted from our recent study [14]. For each "real tree", we employed the tool Bppseqgen v2.4.0 [15] to simulate a sequence alignment under LG amino acid substitution model with Gamma-distributed 10% across-site rate variation. Simulated alignments were then subjected to IQ-TREE v2.0.6 for phylogeny inference with LG+G model and 1,000 ultrafast bootstraps. The bootstrapped trees were then reconciled with the *Prochlorococcus* species tree using the tool ALE v0.4, AnGST v1.0 and ecceTERA v1.2.4, while the IQ-TREE-derived ML tree was used as the initial tree for GeneRax reconciliation. For parsimony-based reconciliations, the costs for gene duplication, gene transfer and gene loss (DTL) events were set to 3, 2 and 1, respectively, which are default values for many parsimony-based reconciliation tools [11, 12, 16] and have been applied in several studies [17, 18]. The qualities of these reconciled gene trees were measured by using the "RF.dist" function in the "phangorn" R package. We found that, for both "real tree" datasets, AnGST had lower mean RF distances and thus outperformed ecceTERA (for MP tools) and GeneRax outperformed ALE (for ML tools) (Fig. S5). We therefore applied AnGST and GeneRax to reconcile the full set of 4,689 *Prochlorococcus* gene family tree with the species tree.

References

1. Hellweger FL, Huang Y, Luo H. Carbon limitation drives GC content evolution of a marine bacterium in an individual-based genome-scale model. *ISME J.* 2018; 12(5):1180-1187.

2. Sung W, Ackerman MS, Dillon MM, Platt TG, Fuqua C, Cooper VS, et al. Evolution of the insertion-deletion mutation rate across the tree of life. *G3 (Bethesda)*. 2016; 6(8):2583-2591.

3. Sun Y, Powell KE, Sung W, Lynch M, Moran MA, Luo H. Spontaneous mutations of a model heterotrophic marine bacterium. *ISME J*. 2017; 11(7):1713-1718.

4. Dillon MM, Sung W, Sebra R, Lynch M, Cooper VS. Genome-Wide Biases in the Rate and Molecular Spectrum of Spontaneous Mutations in *Vibrio cholerae* and *Vibrio fischeri*. *Mol Biol Evol*. 2017; 34(1):93-109.

5. Chen Z, Wang X, Song Y, Zeng Q, Zhang Y, Luo H. *Prochlorococcus* have low global mutation rate and small effective population size. *Nat Ecol Evol*. 2022; 6(2):183-194.

6. Hyatt D, Chen GL, LoCascio PF, Land ML, Larimer FW, Hauser LJ. Prodigal: prokaryotic gene recognition and translation initiation site identification. *BMC Bioinform*. 2010; 11(1):1-11.

7. Yang Z, Nielsen R. Estimating Synonymous and Nonsynonymous Substitution Rates Under Realistic Evolutionary Models. *Mol Biol Evol*. 2000; 17(1):32-43.

8. Kumar S, Nei M, Dudley J, Tamura K. MEGA: a biologist-centric software for evolutionary analysis of DNA and protein sequences. *Brief Bioinform*. 2008; 9(4):299-306.

9. Luo H, Huang Y, Stepanauskas R, Tang J. Excess of non-conservative amino acid changes in marine bacterioplankton lineages with reduced genomes. *Nat Microbiol*. 2017; 2(8):1-9.

10. Szöllősi GJ, Rosikiewicz W, Boussau B, Tannier E, Daubin V. Efficient exploration of the space of reconciled gene trees. *Syst Biol*. 2013; 62(6):901-912.

11. Morel B, Kozlov AM, Stamatakis A, Szöllősi GJ. GeneRax: a tool for species-tree-aware maximum likelihood-based gene family tree inference under gene duplication, transfer, and loss. *Mol Biol Evol*. 2020; 37(9):2763-2774.

12. David LA, Alm EJ. Rapid evolutionary innovation during an Archaean genetic expansion. *Nature*. 2011; 469(7328):93-96.

13. Jacox E, Chauve C, Szöllősi GJ, Ponty Y, Scornavacca C. ecceTERA: comprehensive gene tree-species tree reconciliation using parsimony. *Bioinform.* 2016; 32(13):2056-2058.

14. Zhang H, Sun Y, Zeng Q, Crowe SA, Luo H. Snowball Earth, population bottleneck and *Prochlorococcus* evolution. *Proc R Soc B: Biol Sci*. 2021; 288(1963):20211956.

15. Dutheil J, Boussau B. Non-homogeneous models of sequence evolution in the Bio++ suite of libraries and programs. *BMC Evol Biol*. 2008; 8(1):1-12.

16. Bansal MS, Wu YC, Alm EJ, Kellis M. Improved gene tree error correction in the presence of horizontal gene transfer. *Bioinform.* 2015; 31(8):1211-1218.

17. Nagy LG, Ohm RA, Kovács GM, Floudas D, Riley R, Gácser A, et al. Latent homology and convergent regulatory evolution underlies the repeated emergence of yeasts. *Nat Commun*. 2014; 5(1):1-8.

18. Hehemann JH, Arevalo P, Datta MS, Yu X, Corzett CH, Henschel A, et al. Adaptive radiation by waves of gene transfer leads to fine-scale resource partitioning in marine microbes. *Nat Commun*. 2016; 7(1):1-10.

Fig. S1

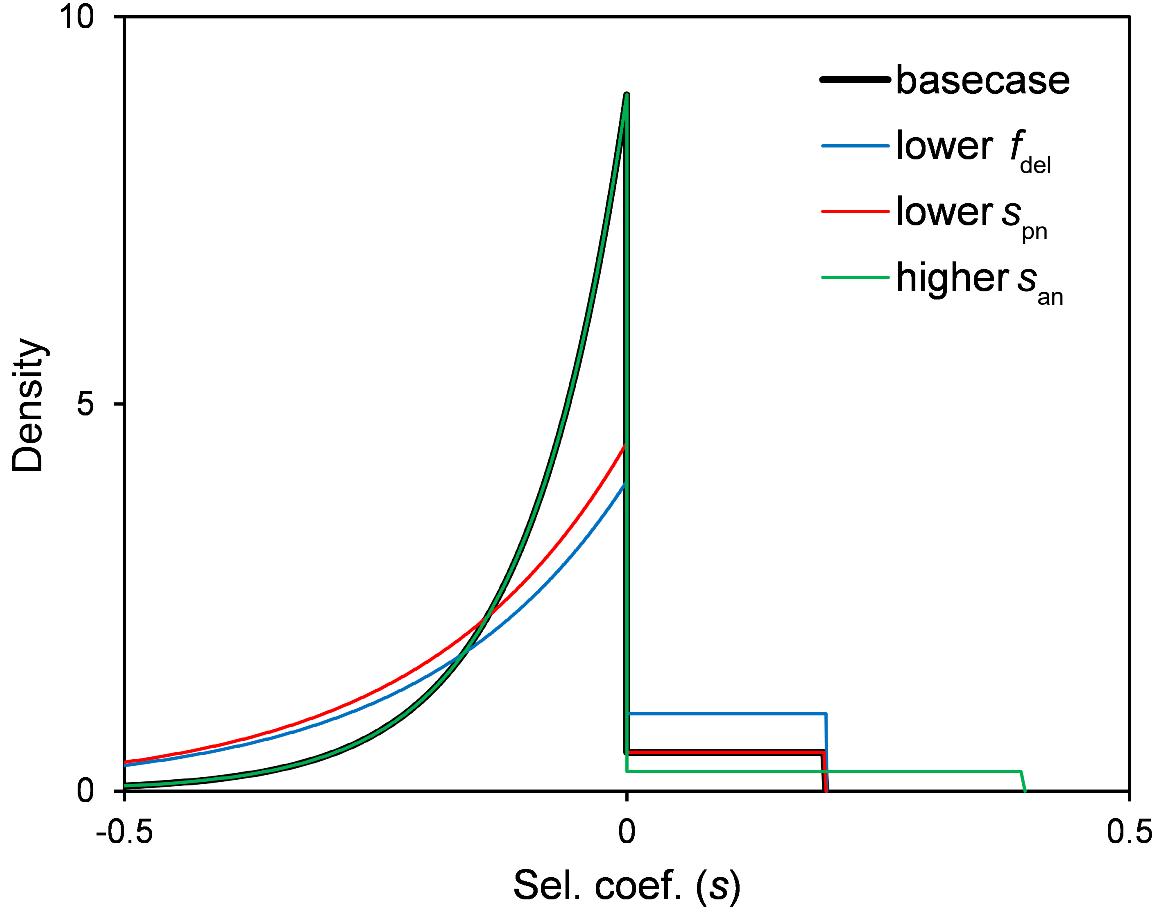

Fig. S1 Nonsynonymous mutation fitness model. Parameters for basecase: f_{del} = 0.9, s_{pn} = -0.1, s_{an} = 0.1. Mutations with larger amino acid chemical distance will have lower s_{pn} values (see details in SI and in Hellweger et al., 2018).

Fig. S2

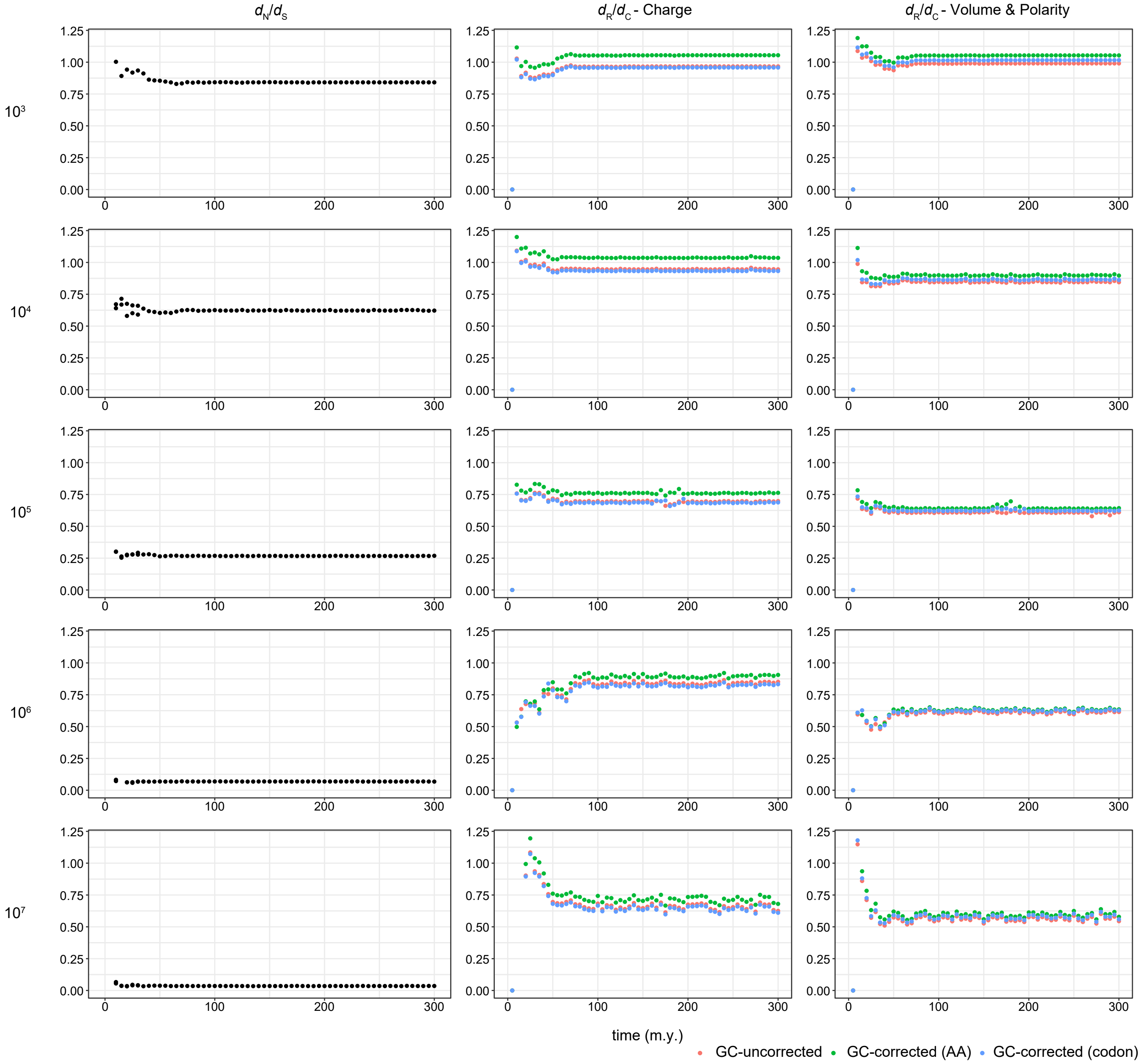

Fig. S2 The genome-wide d_N/d_S and d_R/d_C ratios of simulated *Prochlorococcus* populations in which the genomes were sampled every 5 million years (m.y.). The GC-corrected d_R/d_C values based on codon frequency and AA composition are marked in green and blue, and the uncorrected d_R/d_C values are marked in red.

Fig. S3

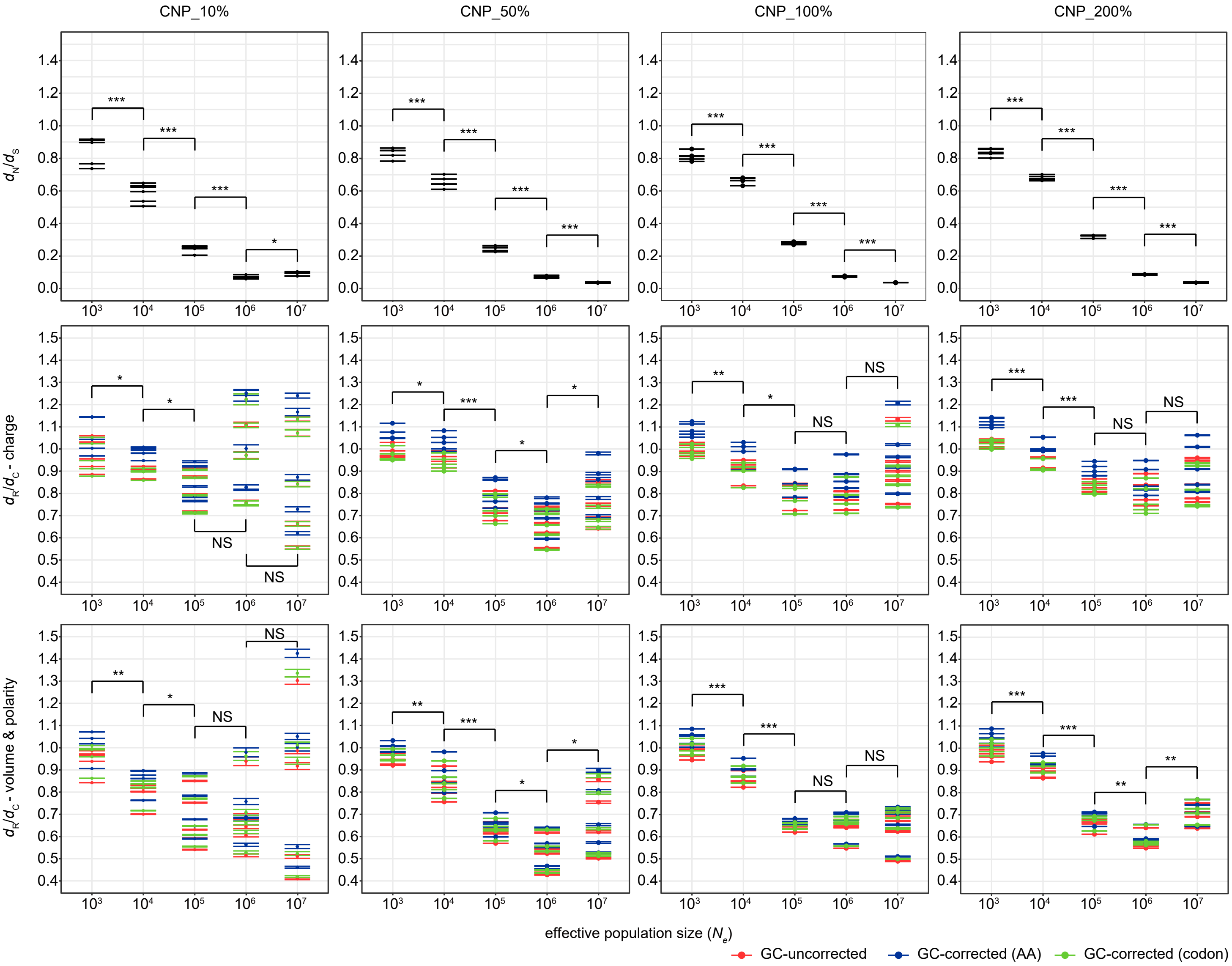

Fig. S3 The genome-wide d_N/d_S and d_R/d_C ratios of simulated *Prochlorococcus* populations under different growth rates. As bacterial growth rate is associated with the concentration of nutrient C, N and P, we controlled the nutrient-associated parameters in our simulations and used CNP_10%, CNP_50% and CNP_200% to represent the tenth, the half and the double growth rate of *Prochlorococcus*. For each N_e, the simulation ran five times and each generated 50 genome sequences. Mean values of the d_N/d_S and d_R/d_C ratios in each run are used for one-tailed t-test (***: $p<0.001$, **: $p<0.01$, *: $p<0.05$; NS: non-significant). The GC-corrected d_R/d_C values based on codon frequency and AA composition are marked in red and blue, and the uncorrected d_R/d_C values are marked in green. The error bars in the plots represent the standard error of the mean (SEM).

Fig. S4

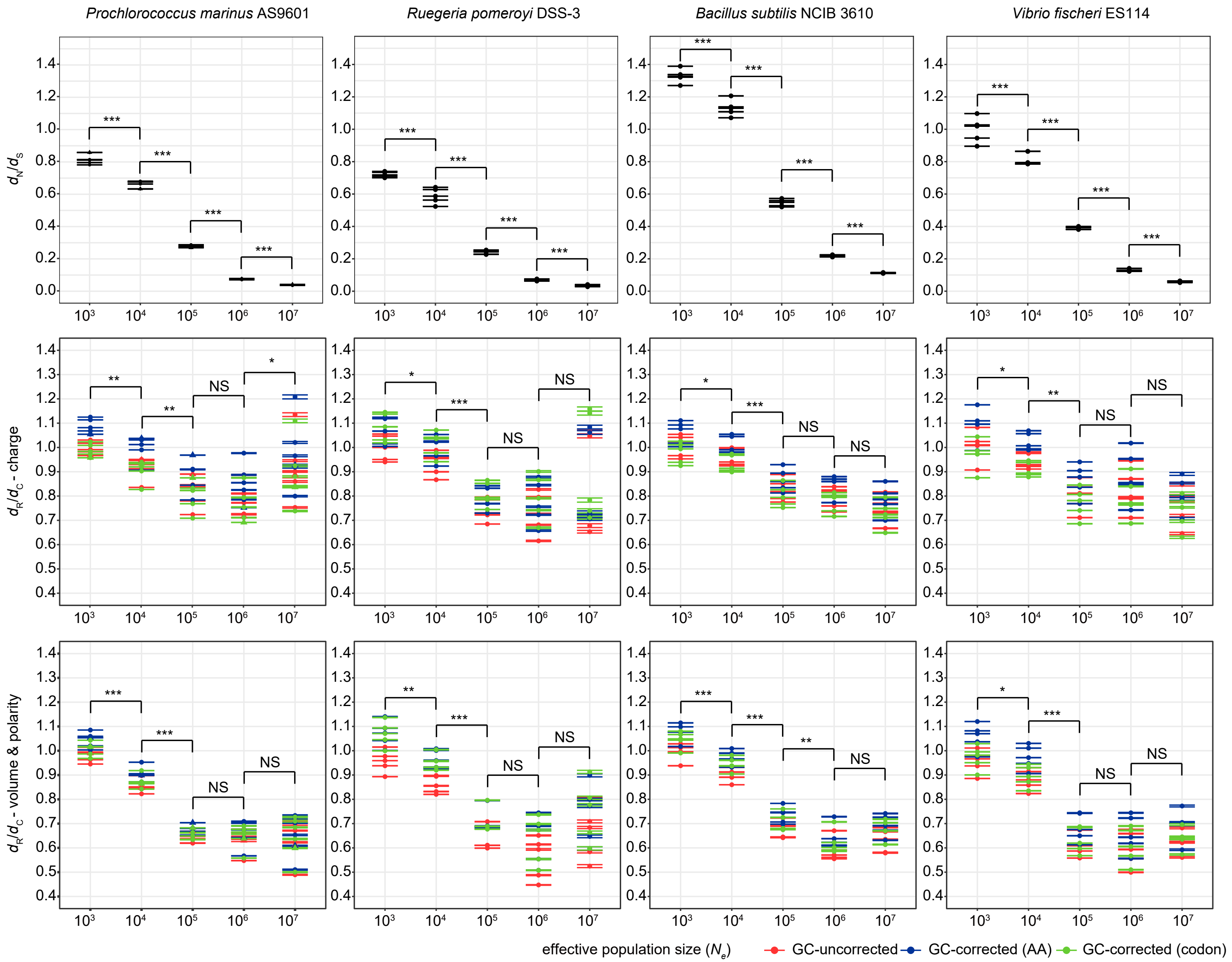

Fig. S4 The genome-wide d_N/d_S and d_R/d_C ratios of simulated populations of *Bacillus subtilis* BSn5, *Ruegeria pomeroyi* DSS-3, and *Vibrio fischeri* ES114. For each N_e, the simulation ran five times and each sampled 50 genome sequences (marked with circle). For *Prochlorococcus marinus* AS9601, an additional simulation run was performed to sample 500 genome sequences for each N_e (marked with triangle). Mean values of the d_N/d_S and d_R/d_C in each run are used for one-tailed t-test (***: $p<0.001$, **: $p<0.01$, *: $p<0.05$; NS: non-significant). The GC-corrected d_R/d_C values based on codon frequency and AA composition are marked in red and blue, and the uncorrected d_R/d_C values are marked in green. The error bars in the plots represent the standard error of the mean (SEM).

Fig. S5

A

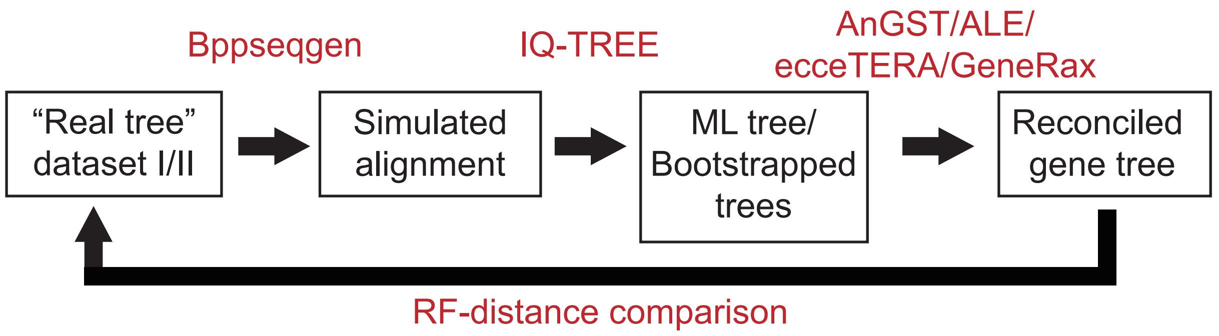

B

RF distance		"Real tree" dataset I		"Real tree" dataset II	
		Mean	SD	Mean	SD
ML	ALE	0.2895	0.0888	0.3452	0.0806
	GeneRax	0.2659	0.0837	0.3319	0.1582
MP	AnGST	0.3457	0.1326	0.4204	0.0935
	ecceTERA	0.4024	0.1674	0.4761	0.1214

C

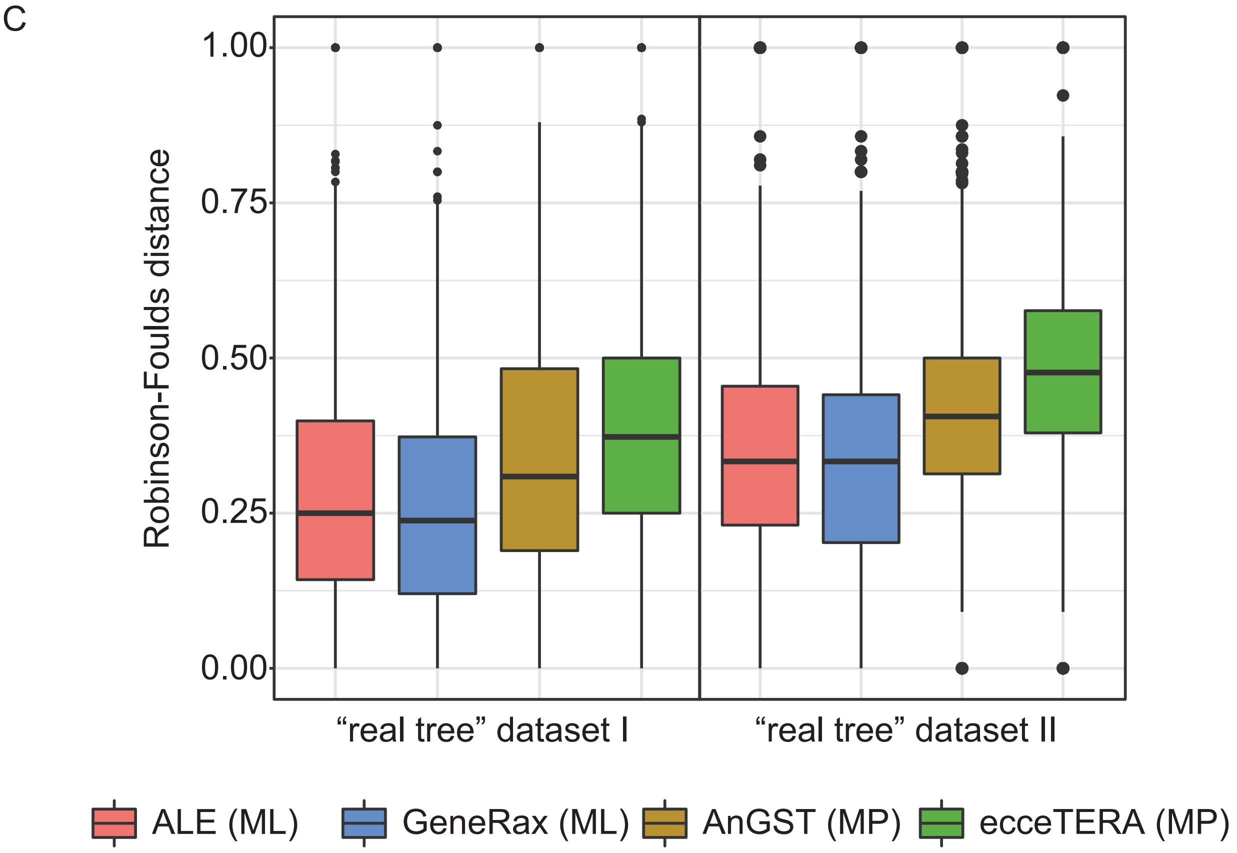

Fig. S5 (A) The illustration of the simulation-based benchmarking workflow, in which the tools are marked in red. (B) The mean and the standard deviation (SD) of the RF distance calculated based on the four reconciliation tools. MP: maximum parsimony-based reconciliation approach; ML: maximum likelihood-based reconciliation approach. The lower RF distance, the higher accuracy of the reconciliation. (C) The boxplot shows the RF distance calculated based on different tools and datasets. The middle line represents the median value of the RF distance. The lower and upper boundary of the box represent the 25th percentile and 75th percentile value of the RF distance, respectively. The upper and lower flank boundary represent the maximum and minimum RF distance, respectively, within the range of median ± 1.5 IQR (interquartile range: 75th percentile value - 25th percentile value). All other RF distance values, which are not included in this range, are marked with dots.
